# Supplementary material for: Are carbon emissions trading and green financial instruments synergistic? -Comprehensive quantitative research based on content analysis
Source: PLoS One. 2024 Mar 7;19(3):e0298601. doi: 10.1371/journal.pone.0298601 (PMC10919601; doi:10.1371/journal.pone.0298601)
Supplement: S1 Data — (ZIP) [file pone.0298601.s001.zip › Data/地级市Z_F工作报告.zip!/░▓╗╒/░÷▓║╩╨/└┤╫╘╣1⁄2╓┌║┼í╛┬φ┐╦╩2╛▌═°í┐_2010.docx]

各位代表： 

　　现在，我代表市人民政府向大会报告工作，请予审议，并请市政协委员和其他列席人员提出意见。

　　一、攻坚克难，锐意进取的2009年

　　2009年是进入新世纪以来我市经济发展最为困难的一年，也是我们经受严峻考验、加快发展步伐的重要一年。一年来，全市各族人民在市委的坚强领导下，深入贯彻落实科学发展观，坚持以保持经济平稳较快发展为首要任务，全力做好保增长、保民生、保稳定的各项工作，较好地完成了市十四届人大三次会议确定的各项目标任务，保持了经济平稳较快增长、社会事业全面进步、人民生活持续改善、社会大局和谐稳定的良好局面。

　　过去的一年，我们坚持危中寻机，抢占先机，不断强化应对国际金融危机的措施，保增长目标如期实现。

　　国民经济平稳较快发展。我们主动作为，远谋近施，认真落实中央和省扩内需、保增长的政策，制定并实施了一系列行之有效的措施，全市经济在应对危机中企稳回升、在破解难题中逆势上扬。预计，全年完成地区生产总值538亿元，同比增长12%以上，其中，一、二、三次产业增加值分别增长5%、17%、10%。规模以上工业增加值185亿元，增长21%。财政总收入75.8亿元，增长15.2%，其中，地方财政收入31.2亿元，增长13.4%。固定资产投资355亿元，增长40.2%。社会消费品零售总额227亿元，增长18.3%。城镇居民人均可支配收入13467元、农民人均纯收入4643元，分别增长6%、8%。单位生产总值能耗降低4.5%。

　　工业经济平稳上行。以保企业、保增长为主线，深入开展“企业服务年”活动，加大资金支持和政策激励，工业强市取得新进展。完成工业投资165.8亿元，柳工汽车起重机一期、三鑫太阳能玻璃等52个项目建成投产，国瑞特重型数控机床、永红电子科技基地等55个项目开工建设。新增规模以上工业企业118户，销售收入亿元以上企业达到76户，增加26户。工业经济效益持续上扬，规模以上工业企业实现利润26亿元，增长20%。园区产业集聚度提高，累计投入175.6亿元，新增入园项目175个。滤清器、不锈钢等特色园区开工建设。

　　农业和农村经济稳步增长。深入推进小麦、水稻、玉米三大粮食生产工程，奋力夺取抗旱保苗全面胜利，粮食总产达264.3万吨，增长4.3%，连续六年创历史新高。提升农业产业化水平，新增市级龙头企业16家、农民专业合作社155个。继续推进规模养殖，新建市级标准化畜禽养殖小区26个。进一步改善农村生产生活条件，完成樵子涧水库除险加固主体工程，基本完成淮北大堤加固等治淮工程，改善防洪面积10万亩，新增、改善有效灌溉面积15万亩，新增绿色长廊31公里、省级林业龙头企业7家，完成成片造林1.5万亩。扶持农民工返乡创业，设立返乡农民工创业园14个。完成粮食清仓查库工作。县域经济发展加快，生产总值、财政收入、规模以上工业增加值增速均高于全市平均水平。

　　服务业规模持续扩大。继续推进“新网”、“万村千乡”、“家电下乡”等工程，新建农家店423个，改造和建设配送中心6个，农村消费市场更加活跃。住房、汽车等消费市场保持旺盛。居民消费价格总水平与上年相比基本持平。不断壮大传统服务业，沃尔玛购物广场、商之都项目建设加快，宝龙城市广场基本完工，光彩奇石玉器城竣工，百大购物中心全面营业。加快发展现代服务业，姜桥物流工业园区、皖北烟叶仓储物流中心等项目加快建设；张公山风景区创建4A级景区通过专家组评估验收；加快地方金融组织建设，8家小额贷款公司开业运营，新建担保公司11家。

　　过去的一年，我们坚持抢抓中央和省扩内需、保增长的政策机遇，加大投入，狠抓招商，推进创新，深化改革，发展后劲和活力进一步增强。

　　固定资产投资成效明显。把握国家投资重点，争取和实施中央新增投资项目301个，落实各类补助资金6亿元。强力实施“3461”行动计划，国电蚌埠电厂一期、固镇伊诺华轮胎制造、五河凯迪生物质发电Ⅰ号机组等45个项目建成投产，翻袋式离心机、淮干河道整治等125个项目开工建设。加强重大项目谋划储备，新增谋划项目90个，总储备规模达528亿元。强化项目融资，与开行签订新一轮合作备忘录，与中行、建行、徽行等商业银行对接合作项目300个。城投公司企业债券发行计划上报国家审批。

　　招商引资势头良好。继续加大招商引资工作力度，积极开展“招商引资月”活动，突出产业转移对接，举办了“北京招商周”、“闽粤浙招商”、“中国·蚌埠2009国际合作暨投资对接会”等招商活动。积极拓展国外招商空间，赴马来西亚和日本招商取得丰硕成果。积极开展与央企合作发展工作，中建材光电产业基地等20个项目签约，投资总额532.2亿元。全年新引进项目559个，成功引进国能生物发电、裕龙机械等一批重大项目；实际利用外资2.68亿美元，引进内资249亿元，分别增长15.5%、38.3%。

　　自主创新建设步伐加快。累计兑现4.56亿元专项资金，重点支持118家企业、196个自主创新项目。大力培育科技创新型企业，新增国家级创新型(试点)企业2家、省级7家，新认定高新技术企业24家。加快创新平台建设，新建省级以上研发平台21个，国家专利技术(蚌埠)展示交易中心、上海理工大学国家大学科技园蚌埠基地投入使用，国家光伏产业研发、生物发酵产业技术等服务平台开工建设。专利授权量380件，增长96%。我市跻身国家知识产权试点市行列。

　　改革开放深入推进。丰原集团改制工作积极稳妥实施，首钢国际成功收购华皖碳纤维。政府机构改革稳步推进。全面推行非税收入和行政事业单位银行账户管理方式改革。完善市对区财政管理体制。启动投融资及城市建设管理体制改革。深化医药卫生、文化、农村综合配套等改革。全面完成工商系统市场管办脱钩工作。拓宽国际交往渠道，与法国布希圣乔治市结为友好交流城市。用足用好外贸促进政策，实现进出口总额3.7亿美元。深入实施全民创业行动，全年新增私营企业1586家、个体工商户9875户。

　　过去的一年，我们坚持加快基础设施建设，强化城市管理，加强节能减排，城市环境继续改善。

　　城市建设和管理得到加强。发挥规划引领作用，编制完成高新区南部拓展区等重点地区控制性详规。大力推进城市基础设施建设，新建改建财大路北段、柳工大道西段、中山街、中荣街等48条道路，完成城市防洪工程新船塘段加固改造，城市南出口改造、大庆路淮河公路桥、高铁新区基础设施等项目开工。张公山大桥桥东绿地、龙子湖桥头公园建设基本完成。大板楼危房改造拆迁安置进展顺利，陶店廉租房一期、太平街棚户区改造工程基本完工。进一步理顺城市管理体制，实行城市长效综合管理日检查、月考评制度，推进管理重心下移，开展占道经营、饮食摊点、交通秩序等重点领域专项整治，市容市貌有新的改观。

　　生态环境不断优化。组织实施环保专项行动，认真落实重点地区、重点行业、重点企业污染治理措施。第二污水处理厂投入试运营，城南污水处理厂前期工作有序推进，改造、新增污水管网20公里。垃圾填埋场渗滤液处理工程开工建设。化学需氧量排放控制在1.9万吨，二氧化硫排放控制在2.71万吨。

　　过去的一年，我们坚持保障和改善民生，统筹发展社会各项事业，和谐社会建设迈出坚实步伐。

　　民生工程积极实施。扎实推进28项民生工程，投入资金12.2亿元，惠及全市城乡居民。承诺的10件实事全面完成。实施积极的就业政策，新增城镇就业人员3.28万人，安置下岗失业人员2.06万人，城镇登记失业率控制在4.5%以内。失业保险市级统筹正式启动。农村低保提标扩面工作基本完成，新型农村合作医疗制度不断完善。城乡医疗救助全面开展，城镇居民基本医疗保险深入推进，贫困重度残疾人和重大传染病病人获得救助。政策性农业保险扎实开展。城乡卫生基础设施和乡镇综合文化站、农家书屋、农民体育健身工程建设加快。广播电视“村村通”工程覆盖率100%。8座小型水库除险加固工程完工，新建13个农村饮水安全工程集中供水处。完成农村公路村村通500公里。开工建设经济适用房17.2万平方米、廉租房19.5万平方米，1.38万户家庭享受到廉租住房保障。新建改造城市公厕10座、维修20座。新购置公交车辆40台，新增及延伸公交线路5条。启动新档案馆、博物馆等规划选址工作。

　　社会事业协调发展。加强薄弱学校建设，实施中小学校舍安全改造工程，完成5.5万平方米校舍加固和新建任务。龙湖教师新村二期开工建设。完善公共卫生服务体系，疾病预防和紧急救治能力全面提升，有效应对手足口病和甲型H1N1流感。加强文化保护与传承，举办双墩遗址刻划符号暨早期文明起源国际学术研讨会等文化活动，双墩一号春秋墓被列入全国十大考古新发现。成功举办《祖国万岁》大型音乐舞蹈史诗、“楚汉杯”全国琵琶大赛等系列国庆活动。龙湖体育馆投入使用。蚌埠籍运动员在全国第十一届运动会上取得优异成绩。妇女儿童发展纲要得到进一步落实。大力推进应急体系建设，新建一批应急疏散避难场所，应急管理工作水平有所提高。全面加强防震减灾工作。取消市区籍车辆通行解放路、朝阳路淮河公路桥收费。完善社会治安防控体系，高度重视安全生产，切实加强信访工作，社会大局保持稳定。人口和计划生育、机关事务管理和政务接待、外事、侨务、老年人、残疾人事业都有新发展，人防、消防、统计、气象、地方志、民族宗教、双拥、国防和后备力量建设等工作取得新成绩。

　　过去的一年，我们坚持执政为民，依法行政，努力提高行政执行力和服务水平，政府自身建设得到加强。

　　我们始终保持奋发有为的精神状态，坚持早安排、快行动、聚合力、强推进，紧盯宏观政策走向，加强经济运行组织与调节，强化政策扶持和服务，完善工作推进机制，政府自身建设在应对金融危机挑战中取得新成绩。认真开展深入学习实践科学发展观活动。完成行政审批服务“两集中、两到位”改革，在全省率先实施行政审批服务标准化建设。取消、停征、降低行政事业性收费117项。启动规范行政处罚自由裁量权试点工作。废止改革开放以来市政府发布的规范性文件109件。认真执行政府信息公开条例，深入推行政务公开和电子政务。自觉接受人大、政协监督，办理人大代表议案7件、建议68件，政协提案370件。政风行风热线、政风行风面对面、市长热线等反映社情民意的桥梁作用进一步发挥。积极推进反腐倡廉制度强化执行年活动，加大行政监察和审计监督力度，严肃查处了一批违纪违法案件。

　　各位代表！在形势异常严峻、发展最为困难的一年，我们能取得这样的成绩，实属不易，这得益于中共蚌埠市委的正确领导和市人大、市政协的有力监督、大力支持，凝聚着全市人民的智慧和汗水，体现了方方面面的支持和帮助。在此，我谨代表市人民政府，向全市各族人民，向广大工人、农民、知识分子、干部和解放军指战员、武警官兵、政法干警，向国家和省驻蚌单位，向各民主党派、工商联、各人民团体和其他各界人士，向人大代表、政协委员，向离退休老同志，向在蚌创业的境内外投资者和建设者，向所有关心、支持和参与蚌埠现代化建设的海内外朋友，表示崇高的敬意和衷心的感谢！

　　在肯定成绩的同时，我们也清醒地认识到我市经济社会发展中还存在一些不容忽视的困难和问题。总体上看发展速度不快，工业化水平较低，长期积累的经济总量不大、产业结构不优、主导产业优势不突出、发展后劲明显不足等矛盾有待进一步解决；重点园区基础薄弱，开发速度和规模还没达到应有水平；县域经济在数量和质量上还没有实现大的突破，农村基础较为落后，乡村经济还没有真正起步；财政收支矛盾突出，税源培育有待加强；城市基础设施建设滞后，城市发展环境有待进一步优化；城乡居民收入增长不快，关系群众切身利益的就业、住房、医疗、教育等问题有待更好解决，城乡社会保障水平需要继续提高。同时，政府职能转变还不适应发展新要求，工作中既存在思想解放程度不够、抓机遇的意识和能力不强的问题，也存在化解矛盾、解决问题、狠抓落实的力度还不够大的现象。对此，我们一定高度重视，并采取切实有效措施加以解决。

　　二、把握形势，明确加快发展的目标任务

　　2010年是完成“十一五”规划的最后一年，也是巩固经济回升基础、谋划“十二五”发展的关键之年。纵观今年发展形势，我们面临的仍然是一个机遇与挑战并存，机遇大于挑战的环境。目前，国际金融危机余波未尽，国内经济回升还存在不确定、不稳定因素。在这样的国际国内宏观形势下，我们不能盲目乐观，必须审慎应对。在看到形势严峻、困难尤存的同时，我们更应该看到加快发展的有利条件和难得机遇。为巩固和增强经济回升向好势头，国家将继续实施积极的财政政策和适度宽松的货币政策，进一步加大城镇建设、战略性新兴产业、“三农”、民生保障等重点领域扶持力度，省委、省政府出台了一系列促进经济平稳较快增长和推动皖北地区加快发展的具体措施，这些都为我市创造了良好的外部环境。从自身来看，随着多年坚持不懈地加大有效投入，发展后劲将进一步增强；随着合芜蚌自主创新综合配套改革试验区建设的全面实施，创新能力将进一步提升；随着投融资及城市建设管理体制等重点领域和关键环节改革的稳步推进，发展活力将进一步显现。面对机遇与挑战，我们必须咬定发展不放松，瞄准目标不动摇，扎实工作，勇往直前，努力保持好、巩固好、发展好经济社会平稳较快发展的势头，力求在推进发展中有大的作为。

　　今年市政府工作的总体要求是：全面贯彻党的十七大和十七届三中、四中全会精神，以邓小平理论和“三个代表”重要思想为指导，深入贯彻落实科学发展观，以继续保持经济平稳较快增长为主要任务，以调整和优化经济结构为战略目标，以深化自主创新和改革开放为重点方向，以项目投资和招商引资为重要抓手，以改善和保障民生为最终目的，抢抓机遇，升级提速，加快推进工业化、城镇化、农业现代化进程，全面完成“十一五”规划目标任务，努力促进经济社会更好更快发展。

　　今年经济社会发展的主要预期目标为：地区生产总值增长12%以上，一、二、三产分别增长5%、17%和11%，规模以上工业增加值增长20%以上；财政总收入增长12%，其中地方财政收入增长13%；固定资产投资增长25%以上；利用境外资金增长10%，引进市外资金增长30%；社会消费品零售总额增长16%；进出口总额增长10%；城镇居民人均可支配收入增长7%；农民人均纯收入增长7%；城镇登记失业率、人口自然增长率、居民消费价格涨幅、化学需氧量排放及二氧化硫排放均控制在省下达的目标以内；单位生产总值能耗降低4.5%。力争主要经济指标增幅达到或超过全省平均水平。

　　以上预期目标，是在全面分析宏观形势，综合考虑各种因素的基础上确定的，总体上说是积极可行的，通过努力也是能够实现的。完成上述目标任务，推进全年工作，需要我们把握好以下几点：一是持续来之不易的好势头。牢牢把握经济社会发展中的积极变化，采取更有针对性的措施，不断夯实经济回升向好的基础，奋力开创经济社会发展新局面。二是持续行之有效的好政策。保持政策的连续性和稳定性，强化政策执行落实，不断丰富、完善和创新政策，做到新变化就有新的应对措施，面对新需要就有新的政策支持。三是持续争先进位的好状态。找准定位，瞄准目标，提升标杆，在攀高比强中加速发展，在争先进位中奋力赶超。四是持续上下齐心的好格局。坚持团结协作、密切配合，多沟通、不争论，多理解、不抱怨，多实干、不折腾，继续把全市人民干事创业的信心和力量凝聚起来，形成推动加快发展的不竭动力。五是持续真抓实干的好作风。敢于较真碰硬，敢于承担风险，善于营造鼓劲氛围，全身心地埋头苦干，把所有精力集中到办实事上，把全部本领用到促发展上。

　　三、团结拼搏，强力推进2010年各项工作

　　2010年，我们要全力以赴积极应对国际国内环境的新变化，紧紧围绕全年目标任务，着重抓好以下八个方面工作：

　　(一)坚持更好更快，努力促进经济增长水平新提升。

　　推动工业提速增效。继续把工业强市作为加快发展的主战略，进一步转变工业增长方式，培植主导产业和骨干企业。一是做强做大工业骨干企业。以企业发展战略为引领，以与央企省企合作发展为抓手，积极引导大企业进行开放式资产重组。重点抓好中粮集团丰原生化生物产品产能扩建、中建材光电产业基地、中电集团第四十一所孵化养殖装备产业化基地等央企合作重点项目，主动做好与省企对接工作。积极实施高科技企业“小巨人”计划，重点培育专精特新中小企业，发展产业集群。鼓励重点企业加大技术引进和技术改造投入，推动企业提高市场竞争力。确保全年新增规模以上工业企业100户，新增销售收入亿元以上工业企业20户。二是推进重点工业项目建设。加快项目前期工作，促成年产260万平方米低辐射玻璃镀膜生产线、年产3万吨聚丙烯酰胺专用化学品等重点项目开工建设；加强项目调度与服务，加快建设永红电子科技基地、巨力全液压智能旋挖钻机等项目，确保安徽首文碳纤维一期技改、玻璃新材料产业园TCO导电膜玻璃等重点项目竣工投产。重点抓好200个千万元以上工业项目，力争工业投资增长50%以上。三是增强园区集聚承载功能。加大开发区基础设施投入，完善物流、生产、生活服务等配套设施。支持开发区扩区工作，拓展园区发展空间。加快市区工业集中区建设，与开发区错位发展。积极探索园区共建新模式，与发达地区、知名企业合作共建开发区。继续推动沫河口化工园、滤清器产业园等各类特色专业园区的规划建设，加快企业退市进园步伐。四是提高工业运行调度和服务水平。健全经济运行的统计分析和监测预警制度；加强煤电油运和资金等生产要素的调度衔接，切实解决企业发展中的实际困难；完善并强化工业发展的政策措施，加大资金支持和政策激励；强化指导和服务，推动企业加强内部管理和市场开拓。

　　切实增加有效投入。保持投资快速增长势头，着力扩大投资规模，改善投资结构，推动项目升级，提高投资效益，确保投资规模超过450亿元，力争达到500亿元。大力推进项目建设。积极实施“3461”行动计划，加大重点项目建设投入，重点项目投资比重占全市固定资产投资40%以上。建成国瑞特重型数控机床、日月电子高档传感器等项目，加快京沪高铁蚌埠段、利用世行和日元贷款等续建项目进度，开工建设合蚌客专蚌埠段、年产800台中低压消防车等项目，推进国电蚌埠电厂二期工程、怀洪新河灌区等项目前期工作。按期完成农村公路改造、市县廉租住房、城镇垃圾污水处理等中央新增投资项目建设任务。积极争取项目和资金。围绕产业结构升级、自主创新、基础设施、社会建设等重点领域，积极编报项目争取上级支持，确保落实各类补助资金达到全省平均数以上。完善项目推进机制。全力开展重点项目专题调度、跟踪服务、施工保障等活动，优化项目建设环境，确保项目顺利推进。强化重大项目谋划储备工作，新增项目谋划储备规模300亿元以上。立足适应新形势、抢抓新机遇、把握新定位、推进新发展，圆满完成“十二五”规划编制工作。

　　加快发展服务业。加强规划引导和政策激励，加快编制现代物流、金融服务等专项规划，制定支持商贸流通业发展政策措施。全面提升传统服务业。完成宝龙城市广场、沃尔玛购物广场、大润发购物中心、光彩二期综合市场等项目建设，加快推进商之都、张公山商业中心、淮河路步行街改造、汇金商业广场等项目建设进度。大力培育现代服务业。拓展提升现代物流业，加快推进姜桥物流工业园区、散装粮食物流配送中心建设，开工建设盐业物流中心，建成皖北烟叶仓储物流中心。加快发展旅游业，积极推进垓下古战场、双墩古迹园、太祖明文化生态产业园、沱湖文化产业园开发建设。大力发展科技服务、广告会展、动漫创意、咨询服务等新兴服务业。积极扩大消费需求。继续推进“新网”、“家电下乡”等工程，拉动农村消费。发挥政策效应，保持房地产市场健康发展。加快发展社区商业、物业服务性消费。整顿和规范市场经济秩序，加强市场监管，依法严厉打击传销活动。

　　(二)加大城乡统筹力度，推动县域经济新发展。

　　大力发展现代农业。着力打造优势农业产业，重点抓好粮食、蔬菜、畜牧标准化养殖小区等建设，落实小麦、水稻、玉米高产核心示范区190万亩，新增市级标准化养殖小区20个。持续推进农业产业化，重点扶持50家农业产业化龙头企业，新增省级龙头企业3家，加快推进50个专业示范村建设，新增农民专业合作社50个，培育市级示范合作社15个。大力实施农业综合开发和科技创新，积极推进龙亢农场现代农业自主创新综合改革试验区、天河现代农业示范项目建设。切实提高农产品质量安全水平，新增农产品认证10个。着力推进淮干河道整治荆山湖行洪区退建工程建设，实施世行贷款重点平原洼地治理工程，新增有效灌溉面积3万亩。新建绿色长廊30公里，完成成片造林1.1万亩。大力发展劳务经济，力争培训新型农民1.5万人以上。加强各项涉农补贴资金和农民负担监管，确保农民负担不反弹。

　　推进城镇化步伐。健全城乡规划体系，完成乡镇建设、村庄布点等规划编制。完善综合交通体系建设，开工建设蚌固一级公路、徐明高速蚌埠段，力争开工建设蚌五高速公路，构建市域快速连接通道。实施县乡通达工程，改造农村公路150公里。以县城和中心镇、中心村建设为重点，加大投入力度，加快道路、供排水、供气、垃圾处理等基础设施建设。扩大县级经济社会管理权限，加大县域项目、用地、金融、人才、技术等扶持力度。大力实施工业强县战略，支持发展特色优势工业、骨干龙头企业，积极培育新型服务业。推进扩权强镇试点，鼓励创建农民创业园，推动农村二、三产业全面发展，着力建设一批经济强镇。全面实施农村土地整治整村推进工作，开展土地整治试点项目建设，打造城镇化和新农村建设的新平台。积极探索城镇户籍制度、社会保障、公共服务等方面改革，建立健全体制机制，引导农民向城镇集中。

　　(三)加强城市建设与管理，打造承载发展新环境。

　　积极开展“建设年”活动，按照主攻基础设施、拉开城市框架、完善城市功能、提升城市品位的要求，在打牢做实城市大建设基础工作方面狠下功夫，着力打造宜业宜居宜游特色鲜明的大城市。

　　加快城市基础设施建设。高起点规划，高标准建设。发挥规划引领城市建设的龙头作用，积极借鉴外地的先进理念和规划定位，提高规划编制、工程建设的水平和质量，提升城市规划编制的品位和档次。优化城市出口道路。完成城市南出口改造，开工建设城市西出口。进一步拉开城市框架。推进实施城市中环线建设，加快建设大庆路淮河公路桥，开工建设解放路和延安路南段、大庆南路等一批城市主干道；继续推进高铁新区、高新区、淮上区道路和龙子湖西岸滨湖新区综合开发、黄山大道建设等。完善城市路网。改建续建道路26条，新开工城市道路25条；加快实施胜利路综合提升改造，基本建成火车站站前道路交通组织项目；继续打通体育路东段、凤阳路西段等一批断头路；改造完成大庆二路、燕山路、友谊路等一批老城区道路。加强公交设施建设，开工建设蚌埠综合客运站等一批公交设施。加强对市区铁路线的整合改造，根据水蚌线电气化改造要求，积极实施水蚌线外迁，开工建设南环铁路线。

　　完善城市公共服务设施。开工建设新档案馆、博物馆、二中新校区、一院综合病房大楼、中医院新院区等一批社会事业项目。基本建成220KV天河、陶山输变电等城市电网工程。积极推进国家玻璃及晶硅材料制品质量检验中心、皖北食品药品检验中心、蚌埠商检中心建设。开工建设城南污水处理厂、第二污水处理厂二期工程，抓好配套污水管网和城市排水管网建设。建成龙子湖、八里沟泵站，完成南施家、新船塘泵站及配套管网建设。继续实施城区天然气、供水管网建设，新增天然气用户2万户，新建供水管网21公里。新建垃圾转运站1座。积极推进城市饮用水水源地安全保障工程。

　　切实改善城市人居环境。实施龙子湖桥头公园北公园二期、东岸综合开发，打造以龙子湖为中心的观光旅游带。加快实施淮河北岸水环境和南岸景观带、张公湖综合环境、席家沟等整治工程。加快创建省级园林城市，实施城市重要街道绿化透绿工程，着力推进城市小街小巷、道路交叉口、城乡结合部除荒补绿工程，开展“百万人种百万棵树”活动，新增城市绿地155公顷。加快棚户区和旧住宅区改造，开工建设滨河西片区、钓鱼台等15个棚户区，开工面积达到20万平方米以上，积极实施山香村等“城中村”改造。加快廉租房建设步伐，完成陶店廉租房二期工程，新开工建设廉租房25万平方米、经济适用房10万平方米。

　　三县也要结合实际，积极开展“建设年”活动，力争县城基础设施建设有新加强、发展环境有新优化、居民生活质量和公共服务水平有新提升。

　　提升城市管理水平。加强市容环境卫生管理，继续抓好货运三轮车、无照小餐饮、小区物业环境等七项专项整治活动；加强对四小车辆、非法广告、占道经营、建筑渣土、油烟噪音污染等专项整治；突出建筑物立面出新、广告提升、示范街区三个重点，加快城市长效综合管理由平面整治向立体整治推进、由主干道路向小街巷推进、由硬化净化向美化亮化推进，力争市容市貌有较大改观，努力实现两年明显见效的目标。进一步完善城市管理综合考评与奖惩机制，探索实施市容数字化、网格化管理模式。加大拆违工作力度，严格控制新生违法建筑。积极开展丰富多彩的文明创建活动，巩固提升文明创建成果。

　　(四)推进自主创新建设，力促经济结构调整新突破。

　　不断增强自主创新能力。加快实施创新型企业培育计划，鼓励企业加大研发投入，积极建设起重机研究院、医药工程实验室等研发平台，推动市级工程技术研究中心、企业技术中心建设，培育省级以上研发平台15个。开展多种形式的产学研合作，重点支持光伏、生物等领域共性技术和关键技术研发，组织实施玉米芯生产聚乳酸、小丝束高强度碳纤维研发等8项重大科技攻关项目。推进创新平台建设，充分发挥国家专利技术蚌埠展示交易中心、上海理工大学国家大学科技园蚌埠基地等创新服务平台作用，加快大学科技园建设。提升知识产权创造、运用、保护和管理能力，全年专利申请量达到1000件。

　　加快产业结构优化升级。邀请国际知名专业机构编制城市功能定位、主导产业选择及产业空间布局规划，加快编制九大工业重点产业规划，充分发挥规划对产业发展的引领作用，促进产业集聚升级。建立产业配套机制，加快装备制造等产业配套发展，做强做大优势产业；建立产业扶持机制，促进新能源、生物、新材料等产业跨越式增长，培育壮大新兴产业，力争六大创新型产业完成投资150亿元，年产值达400亿元；建立产业牵动机制，积极引导发展精深加工，着力激发纺织、建材、轻工等产业发展活力，改造提升传统产业；落实扶持高新技术企业发展的优惠政策，大力发展高新技术产业。

　　加强环境保护和节能减排。强化对年耗能5000吨标煤以上大户的节能监管，积极开展资源综合利用产品认定工作，坚决淘汰落后产能。认真落实饮用水源保护措施，确保饮水安全。严格建设项目环境准入，落实环境保护“三同时”制度，大中型建设项目环评执行率达95%以上。继续开展废水、固废、大气、噪声及农业面源和畜禽养殖污染等防治工作。

　　(五)深化改革开放，激发跨越崛起新动力。

　　推动招商引资升温加压。创新招商引资体制机制，建立重点招商引资项目定期调度制度，开展项目互查和第三方检查，增强招商引资实效。加大招商引资工作奖惩力度，严格落实领导责任制和责任追究制，确保招商引资总额突破350亿元，向400亿元迈进。精心组织重大招商活动，围绕重大产业和重点企业，突出产业招商和专题招商，开展“第三届中国花鼓灯歌舞节暨蚌埠投资贸易洽谈会”、“上海经贸对接会”、“广东产业转移对接会”等招商引资活动，全年安排组织招商活动30次以上。注重招商引资信息收集、项目跟踪，建立招商引资项目信息升级接力机制，明确专业部门对项目进行跟踪落实。加强招商引资机构和队伍建设，建立健全招商网络，提高招商能力。

　　积极稳妥实施各项改革。完善市属国有企业改革工作，稳妥处置改制企业遗留问题，加快推进县属区属企业改革。完成县区政府机构改革。全面实施事业单位绩效工资制度。完善政府预算体系，强化政府非税收入管理，优化财政收支结构。深化农村综合配套改革。加快推进城市建设管理、医疗卫生、教育、文化等体制机制改革。

　　推动新一轮全民创业。积极搭建全民创业载体和服务平台，加快全民创业基地、创业园区和返乡农民工创业园、县区创业孵化中心建设，提升创业辅导中心服务水平。继续落实全民创业扶持政策，完善激励机制，及时解决创业者在项目、资金、土地、技术等方面的实际问题。新增个体工商户10000户、私营企业1500家。

　　提升对外开放水平。坚持市场多元化战略和品牌战略，扶持丰原生化、八一化工等企业进一步开拓国际市场，扩大对外贸易规模，努力保持出口稳定增长。支持丰原集团、安徽水利等企业“走出去”。主动参与泛长三角区域分工合作，全方位承接产业转移。充分发挥区位、资源和产业优势，积极参与淮海经济区等区域协调发展。深化友好城市合作，扩大对外交流成果。

　　(六)拓宽融资渠道，提高金融服务新水平。

　　扩大有效信贷。落实和完善金融政策，支持、引导金融机构加大信贷资金投放额度。继续加强政银企合作交流，探索建立“金融超市”、“企业融资服务平台”等新型银企对接模式。鼓励金融创新，切实破解中小企业、个体工商户和农户融资难题。

　　增强融资能力。深化投融资体制改革，做大做强投融资平台，加大政府性融资力度。落实好开行13个项目贷款资金，积极争取农发行及各商业银行城市基础设施项目贷款。继续推进城投公司20亿元债券发行工作。积极推动直接融资，培育泰格生物、安徽昊方、天润化工、普乐新能源等上市辅导企业，力争1-2家企业上市，鼓励佳先股份等企业开展股权交易融资。

　　发展金融组织。整合壮大担保机构，完成首家村镇银行筹建工作，力争组建1家农村合作银行、引进1家股份制商业银行。积极推进小额贷款公司试点工作。健全政策性农业保险制度，鼓励保险公司发展农业和农村小额保险，扩大农业保险覆盖范围。

　　优化金融环境。建立信息互通、重大事项通报制度，健全信息交流沟通平台。继续加大金融经济秩序整顿力度，支持化解银行业金融机构不良贷款和债务，严厉打击非法金融活动，积极防范和化解金融风险，为金融机构发展营造良好环境。

　　(七)切实加强民生保障，实现群众生活新改善。

　　多渠道多形式增加就业。完善和落实积极的就业政策，确保城镇新增就业3.1万人。大力推进创业带动就业工程，落实小额担保贷款等创业优惠政策。加强劳动职业技能培训，完成培训2.5万人次。健全功能完善、便民高效的就业服务网络，大力开发公益性岗位，动态消除城市零就业家庭。

　　加快完善社会保障体系。做好城镇养老保险省级统筹实施工作。健全被征地农民养老保险制度，加快推进新型农村社会养老保险试点。巩固城镇居民基本医疗保险全覆盖工作，逐步实施市级统筹。积极做好失业保险市级统筹工作。继续推动参保扩面和基金征缴工作，实现征缴率90%以上。强化社保基金监督管理，确保基金安全运行。逐步健全社会救助体系，保障困难群体基本生活。

　　巩固提升民生工程。进一步调整财政支出结构，实施好33项民生工程，把更多的财力向基层倾斜、向民生倾斜、向困难群体倾斜，让全市人民得到更多实惠。提高城乡居民最低生活保障人均补差水平，加快农村五保供养服务机构建设，完善城镇未参保集体企业退休人员基本生活费保障机制，进一步提高群众生活保障水平。实施农村饮水安全工程，解决16万农村人口饮水安全问题。对城市低保住房困难家庭应保尽保，推进50万平方米保障性住房建设，进一步加大住房保障力度。免除城乡义务教育阶段学生学杂费，提高农村贫困寄宿生生活费补助标准和农村义务教育阶段中小学生均公用经费标准，进一步保障学生受教育权利。实行市辖区新农合工作市级统筹管理，完善重大传染病病人医疗救治和艾滋病病人生活救助保障机制，加快城乡卫生基础设施建设，完成5所乡镇卫生院、111所村卫生室、27个社区卫生服务中心(站)标准化建设和基层医疗卫生机构综合改革试点工作，进一步提高群众健康水平。加快推进农村公路、渡改桥工程建设，进一步解决农村群众出行不便问题。加快农家书屋、乡镇综合文化站、农民体育健身工程建设，进一步丰富群众文化生活。落实好中小学校舍安全工程、光荣院建设、农村留守儿童之家建设等5项新增民生工程。

　　民有所呼我有所应，民有所需我有所为。在全力实施民生工程的同时，继续为群众再办10件实事：实施新型农村合作医疗IC卡制度，实现市辖区参合农民就诊“一卡通”；帮助1000名有劳动能力的残疾人实现就业；深入推进万名银发退休困难人员帮扶服务工程；启动室内全民健身活动中心建设；开工建设高新区公交枢纽站、解放路公交枢纽站；新购置公交车辆90台，新增及改造公交线路3条；实施街巷小区路灯便民工程，完成80条小街、117个小区路灯安装；开展城区背街小巷乱搭乱建整治工程；建成医疗废物集中处置中心；完成社会治安视频监控系统建设。

　　(八)完善基本公共服务，推进和谐社会建设新进展。

　　加快各项社会事业发展。继续实施中小学布局优化调整，推进义务教育阶段学校标准化建设工作。深入实施素质教育，深化教育教学改革，全面提高学生综合素质。全面加强公共卫生服务体系建设，完善重大疾病防控措施，深入开展爱国卫生运动。加大文化艺术精品创作力度，广泛开展群众性文化活动。继续开展禹墟、垓下等考古发掘和文物保护工作。加快发展广电事业，建成广播电视塔并投入使用，推进数字电视整体平移工程。积极推动全民健身，力争在省第十二届运动会上实现金牌“保二”任务。继续做好人口和计划生育、机关事务管理和政务接待、外事、侨务、民族宗教、对台、防震减灾工作，重视和推动地方志、气象、红十字会、妇女儿童、未成年人保护、老龄和残疾人等事业发展。

　　维护社会和谐稳定。严格落实信访责任制，继续规范信访秩序。扎实做好安全生产工作，坚决遏制重特大事故的发生。健全质量安全检测监控体系，深入开展食品药品等专项整治。加强应急管理平台建设，提升突发事件应对和处置能力。强化社会治安综合治理，严厉打击各类犯罪活动，全力维护国家安全和社会稳定。积极开展双拥共建活动，认真做好国防动员、民兵预备役和人防工作。

　　各位代表，今年的任务很重，要求更高，要完成新的一年经济社会发展各项目标任务，必须切实加强政府自身建设，以更宽的视野和创新的思维谋求更好更快地发展。

　　切实转变职能，提升服务发展的干事水平。加快推进政府职能转变，更加注重履行社会管理和公共服务职能，不断提高行政效能和服务水平。完善目标管理考核办法，强化督查落实，加强执行监督，确保政令畅通，推动各项决策落到实处。

　　提高综合素质，增强依法行政的干事能力。加强学习调研，力避“本领恐慌”，建设学习型政府。严格按照法定权限和程序行使职权、履行职责，自觉接受人大、政协和社会各界的监督，认真办理人大代表议案、建议和政协提案。积极推行政务公开，完善重大事项决策、专家咨询、社会公示听证等制度，切实提高政府透明度和公信力。

　　坚持以民为本，强化为民谋福祉的干事意识。建立健全为民办实事的长效机制，切实把实现群众的愿望、满足群众的需要、维护群众的利益，作为我们一切工作的出发点和落脚点，把人民的利害得失放在第一位，把人民的安危冷暖放在第一位，把人民的所期所盼放在第一位，以崇高的责任感和使命感履行宗旨、实践宗旨，尽心竭力解决涉及群众切身利益的热点难点问题，真正让广大群众共享改革发展的成果。

　　严格遵守法纪，树立勤政廉洁的干事形象。全面落实党风廉政建设责任制，深入推进教育、制度、监督并重的惩治和预防腐败体系建设。加大对建设工程招投标、经营性土地使用权出让等重要领域监管力度，强化对重点建设资金、重大建设项目审计督查。牢固树立过紧日子的思想，坚持把有限的财力用在发展经济、改善民生上。

　　各位代表，新的一年，赋予我们新的重任、也蕴含新的机遇；新的一年，充满新的挑战、更充满新的希望。让我们以邓小平理论和“三个代表”重要思想为指导，深入贯彻落实科学发展观，在中共蚌埠市委的坚强领导下，团结带领全市各族人民坚定信心，鼓足干劲，迎难而上，再创佳绩，为全面完成“十一五”规划和今年各项目标任务，推动全市经济社会更好更快地发展而努力奋斗！
